# Solving the Traveling Salesman's Problem Using the African Buffalo Optimization

**DOI:** 10.1155/2016/1510256

**Published:** 2016-01-10

**Authors:** Julius Beneoluchi Odili, Mohd Nizam Mohmad Kahar

**Affiliations:** Faculty of Computer Systems & Software Engineering, Universiti Malaysia Pahang, 26300 Kuantan, Malaysia

## Abstract

This paper proposes the African Buffalo Optimization (ABO) which is a new metaheuristic algorithm that is derived from careful observation of the African buffalos, a species of wild cows, in the African forests and savannahs. This animal displays uncommon intelligence, strategic organizational skills, and exceptional navigational ingenuity in its traversal of the African landscape in search for food. The African Buffalo Optimization builds a mathematical model from the behavior of this animal and uses the model to solve 33 benchmark symmetric Traveling Salesman's Problem and six difficult asymmetric instances from the TSPLIB. This study shows that buffalos are able to ensure excellent exploration and exploitation of the search space through regular communication, cooperation, and good memory of its previous personal exploits as well as tapping from the herd's collective exploits. The results obtained by using the ABO to solve these TSP cases were benchmarked against the results obtained by using other popular algorithms. The results obtained using the African Buffalo Optimization algorithm are very competitive.

## 1. Introduction

The growing need for profit maximization and cost minimization has never been greater in human history than what we have today. This need has made optimization a very favoured area of scientific investigations. This development has led to the design of a number of optimization algorithms. Some of the most popular algorithms are the Particle Swarm Optimization [1], Ant Colony Optimization [2], Genetic Algorithm [3], Artificial Bee Colony [4], and many others. However, the above algorithms do have some drawbacks ranging from premature convergence [5], delay in obtaining results, easily being stuck in local minima, and complicated fitness function to having many parameters that require setting up [6]. An attempt to proffer solutions to some of the weaknesses of these algorithms is the motivation for the development of the African Buffalo Optimization (ABO).

The ABO is a population-based stochastic optimization technique that has its inspiration from the behaviour of African buffalos: a species of wild cows, similar to their domestic counterparts that navigate their way through thousands of kilometres in the African rain forests and savannahs by moving together in a large herd of, sometimes, over a thousand buffalos. Their migration is inspired by their search for lush grazing lands. They tend to track the movement of the rainy seasons when they can get lush grazing pastures. As the seasons differ from location to location in the vast African landscape, the buffalos are always mobile in pursuit of their target pastures. In ABO, our interest is in how the buffalos are able to organize themselves in searching the solution space with two basic modes of communications, that is, the alarm “waaa” sounds to indicate the presence of dangers or lack of good grazing fields and, as such, asking the animals to explore other locations that may hold greater promise. On the other hand, the alert “maaa” sound is used to indicate favourable grazing area and is an indication to the animals to stay on to exploit the available resources.

*The Traveling Salesman Problem*. The Traveling Salesman's Problem (TSP) is the problem faced by a salesman who, starting from a particular town, has the assignment of finding the shortest possible round trip through a given set of customer towns or cities. The salesman has a mandate to visit each city once before finally returning to the starting town/city. The Travelling Salesman's Problem (TSP) can be represented by a complete weighted graph *G* = (*V*, *E*) with *V* being the set of *n* nodes (cities) and *E* being the set of edges fully connecting the nodes in the graph *G*. In this graph, each edge *E* is given a weight *d*_*ij*_ which represents the distance between cities *i* and *j*. It may be important to emphasize that the distance between towns/cities may be symmetric (where the distances between the cities are the same in either going or returning from the towns/cities) or asymmetric (where due to some restrictions, possibly, due to one-way lanes or other reasons, the distances of going from city *A* to city *B* may not be the same). The basic minimization equation for TSP is given *n* towns and their coordinates, find an integer permutation *π* = *C*_1_, *C*_2_, *C*_3_,…, *C*_*n*_ with *C*_*n*_ being the city “*n*”, our task is to minimize the sum of the cities [7, 8]. Consider

(1) $$\begin{array}{c} f\left(\;\pi \;\right)=\overset{n-1}{\underset{i=1}{\sum }}d\left(\;C_{i},C_{i+1}\;\right)+d\left(\;C_{n},C_{1}\;\right). \end{array}$$

Here, *d*(*C*_*i*_, *C*_*i*+1_) represents the distance between city “*i*” and city *i* + 1 and *d*(*C*_*n*_, *C*_1_) is the distance of city “*n*” and city “1.” The aim is to find the shortest path between the adjacent cities in the form of a vector of cities.

This paper is organized in this way: Section 1 introduces the African Buffalo Optimization and the Travelling Salesman's Problem; Section 2 presents a brief review of relevant literature; Section 3 discusses the basic flow of the African Buffalo Optimization (ABO) algorithm; Section 4 is concerned with how the ABO solves the TSP; Section 5 deals with the experiments and discussion of the results of the symmetric TSP instances; Section 6 is concerned with the experiments on the asymmetric TSP instances and the discussions of the results; Section 7 examines the performance of ABO vis-à-vis Neural Networks methods; Section 8 draws conclusions on the study.

## 2. Literature Review

Nature-inspired algorithms (NAs) draw their inspiration from the complex but highly organised attributes of nature. Nature-inspired algorithms which are generally stochastic became the hotbed of several researchers due to the inefficiencies of the exact optimization algorithms (as the problem size enlarges) like the Linear Programming, [9], Dynamic Programming [10], finite elements [11], and finite volume methods. In general NAs simulate the interplay and, sometimes, interdependence of natural elements such as plants, animals, and rivers. The most popular class of algorithms among the NAs are the biology-inspired algorithms. A handful of other NAs, however, are inspired by Chemistry or Physics. Some of those algorithms inspired by Chemistry or Physics include Harmony Search (HS) algorithm, Intelligent Water Drop (IWD), Simulated Annealing (SA), and Black Hole (BH) [12]. In this study, our interest is the biology-inspired optimization algorithms.

Biology-inspired algorithms (BAs) can be categorised into three broad classes, namely, Evolutionary Algorithms (EAs) that are inspired by natural evolutions, the Swarm Intelligence (SI) which simulates the collective behavior in plants and animals, and, thirdly, Ecology algorithms which are concerned with simulating the inter- and intracooperative or competitive interactions within the ecosystem [13].

The Evolutionary Algorithms (EAs), generally, simulate the iterative progress comprising growth, development, reproduction, selection, and survival as seen in a population. EAs are concerned with the feasible solutions that emanate iteratively from generation to generation towards the best solution. EAs employ fitness-based selection within the population of solutions in a way that fitter solutions are selected for survival into the next generation of solutions. In this category of EAs are Genetic Programming (GP), Paddy Field Algorithm (PFA), Evolutionary Strategies (ES), Genetic Algorithm (GA), and the Differential Evolution (DE) [12]. It may be necessary to state that experts have different opinions on the classification of Differential Evolution as an Evolutionary Algorithm. The classification of DE as an Evolutionary Algorithm stems from its use of “Evolution” as one of its parameters (just like other EAs). A critical expert may not class Differential Evolution as being bioinspired, actually [14].

The second category of BAs is the Swarm Intelligence (SI) algorithms which are concerned with the collective social behavior of organisms. The motivation of Swarm Intelligence is the collective intelligence of groups of simple agents such as insects, fishes, birds, bacteria, worms, and other animals based on their behavior in real life. As simple as these animals are, they are able to exhibit exceptional intelligence whenever they work collectively as a group. These algorithms track the collective behavior of animals that exhibit decentralized, self-organized patterns in their foraging duties. Examples of these algorithms are the Bee Colony Optimization (BCO), Firefly Algorithm (FFA), Particle Swarm Optimization (PSO), Ant Colony Optimization (ACO), Artificial Bee Colony (ABC), Bacteria Foraging Algorithm (BFA), and so on [15].

The third category of BAs is the Ecology algorithms which are concerned with the numerous inter- or intraspecies competitive or cooperative interactions within the natural ecosystems. The ecosystem is made up of living organisms along with their abiotic environment with which the organisms interact such as water, air, and soil. Cooperation among organisms includes division of labor and represents the core of their sociality. Some of the interactions are cooperative and others are competitive leading to a complex and harmonious balancing within the ecosystem. Algorithms in this category are the PS2O, Invasive Weed Colony Optimization, and biogeography-based optimization [16].

The development of the African Buffalo Optimization (ABO) is in response to the observed lapses in the existing algorithms. The ABO belongs to the Swarm Intelligence class of algorithms based on the social behavior in animals with the aim of achieving greater exploitation and exploration of the search space, ease of use, and faster speed in achieving the optimal result with its use of relatively fewer parameters in solving combinatorial optimization problems.

### 2.1. Ant Colony Optimization (ACO)

Ant Colony Optimization algorithm is a population-based optimization technique developed by Marco Dorigo and has been successfully applied to solve several NP-hard combinatorial optimization problems. This algorithm was inspired by the behavior of ant colonies, especially, by their foraging behavior in real life. Usually ants, when leaving their nests, move randomly around the areas surrounding their nests in search for food. If any of the ants come across food, it first collects some pieces of the food and, on its way back to the nest, deposits a chemical substance called pheromones as a way of communicating to its peers that there has been a breakthrough. Other nearby ants, on perceiving the fragrance of the pheromone, understand and move towards the pheromone path. Once they discover the food source, they, in turn, drop fresh pheromones as a way of alerting other ants. In a matter of time, several ants pick this information and are on the pheromone path.

Another interesting part of the ants' behavior is that as they return to the nest, they optimize their route. In a short while, the ants have created a shorter route to the food source than the previous routes. Moreover, in case an obstruction is put on the shorter route, making movements impossible, the ants are able to find another short route among the available options to evade the obstacle. The highlights of this algorithm include tapping into the indirect communication of a colony of (artificial) ants using pheromone trails as a means of communication, tracking their cooperative ability to solve a complex problem, and harnessing their capacity to optimize their routes from the food source to the nest and vice versa.

There have been several modifications of the ant colony algorithms starting from the initial Ant System (AS), to Ant Colony System (ACS), to Min-Max Ant System (MMAS), and then to the Ant Colony Optimization (ACO) algorithms, and so forth [17]. In ACO, a colony of ants in each iteration constructs a solution probabilistically as ant *k* at node *i* selects the next node*j* to move on to. The choice of node is influenced by the pheromone trail value *τ*_*ij*_^.^(*t*) and the available heuristic *η*_*ij*_. In TSP, *η*_*ij*_ = 1/*d*_*ij*_. So an ant moves from location *i* to location *j* with the probability

(2) $$\begin{array}{c} P_{ij}^{t}\left(\;t\;\right)=\left\{\begin{array}{cc} \frac{{\left\lceil \tau_{ij}^{.}\left(t\right)\right\rceil }^{\alpha }x{\left[\eta_{ij}^{.}\right]}^{\beta }}{\sum_{l\in N_{i^{k}}\left[\tau_{ij}\left(t\right)\right]}\alpha x{\left[\eta_{ij}\right]}^{\beta }} & \text{if }\;j\in N_{i^{k}},\\ 0, & \text{otherwise.} \end{array}\right. \end{array}$$

Here, *τ*_*ij*_^.^(*t*) represents the pheromone trail, *η*_*ij*_ represents the local heuristic information *t* represents the iteration, *𝒩*_*i*^*k*^_ represents the nodes ant *k* can go to, and *α* and *β* are parameters that bias the pheromone trails. By the end of an iteration, the pheromone trail on each edge *ij* is updated using the following equation:

(3) $$\begin{array}{c} \tau_{ij}^{.}\left(\;t+1\;\right)=\left(\;1-\rho \;\right)x\tau_{ij}^{.}\left(\;t\;\right)+{\Delta \tau_{ij}^{.}}^{best}\left(\;t\;\right). \end{array}$$

In (3), *τ*_*ij*_^.^(*t* + 1) represents the pheromone trail in iteration *t* + 1; *ρ* takes any values from 0.1 to 0.9. The amount of pheromone deposited by the best ant is represented by

(4) Δτij.bestt=1fsbesttif  the  best  ant  used  ij  in  iteration  t0,otherwise.

In (4), *f*(*s* ^best^(*t*)) represents cost of the best solution (*s* ^best^(*t*)).

A critical examination of the Ant Colony Optimization technique of solving optimization problems reveals that there is little or no similarity between the ACO's search mechanism and that of the ABO. This could be due to their application of different search techniques in arriving at solutions: while the ACO employs path construction technique, the ABO favours path improvement search mechanism.

### 2.2. Particle Swarm Optimization

Particle Swarm Optimization which was inspired by the social behavior of birds flocking or fish schooling is one of the biology-inspired computation techniques developed by Eberhart and Kennedy [18]. This algorithm obtains solutions using a swarm of particles, where each particle represents a candidate solution. When compared to evolutionary computation paradigms, a swarm is similar to a population and a particle represents an individual. In searching for a solution, the particles are flown through a multidimensional search space, where the position of each particle is adjusted according to its own experience and that of its neighbors. The velocity vector drives the optimization process as well as highlighting each particle's experience and the information exchange within its neighborhood. Just like the ABO, PSO has two controlling equations in its search for solution and these are (5) and (6). Consider

(5) $$\begin{array}{c} v_{ij}^{t+1}=x\left(\;v_{ij}^{t}+\emptyset_{1}U_{1}\left(\;b_{ij}^{t}-x_{ij}^{t}\;\right)+\emptyset_{2}U_{2}\left(\;b_{\left(n\right)ij}^{t}-x_{ij}^{t}\;\right)\;\right), \end{array}$$

where *v*_*ij*_^*t*+1^ represents the present velocity, *v*_*ij*_^*t*^ is the previous velocity, *x* is the constriction factor, *∅*_1_ and *∅*_2_ are the acceleration coefficients, *U*_1_ and *U*_2_ are random numbers, *b*_*ij*_^*t*^ is the individual particles' best position, *x*_*ij*_^*t*^ is the present position, and *b*_(*n*)*ij*_^*t*^ is the swarm's best position. The next equation in PSO that calculates the position of the swarm is

(6) $$\begin{array}{c} x_{ij}^{t+1}=x_{ij}^{t}+v_{ij}^{t+1}. \end{array}$$

In PSO algorithm, the particles move in the domain of an objective function *f* : Θ ∈ *Rn*, where *n* represents the variables to be optimized. Each particle, at a particular iteration, is associated with three vectors:

(a) Present position, denoted by *x*: This vector records the present position of a particular particle.
(b) Present velocity, denoted by *v*: This vector stores the particle's direction of search.
(c) Individual's best position, denoted by *b*: This vector records the particular particle's best solution so far since the search began (since the beginning of the algorithm's execution). In addition to these, the individual particles relate with the best particle in the swarm which PSO algorithm tracks, in each iteration, to help direct the search to promising areas [19].

### 2.3. Artificial Bee Colony

This algorithm, which is inspired by the behavior of natural honey bee swarm, was proposed by Karaboga and Akay in 2009 [20]. It searches for solution through the use of three classes of bees: scout bees, onlooker bees, and employed bees. Scout bees are those that fly over the search space in search for solutions (food source). The onlooker bees, on the other hand, are the ones that stay in the nest waiting for the report of the scout bees while the employed bees refer to the class of bees which, after watching the waggle dance of the scout bees, opts to join in harvesting the food source (exploitation). A particular strength of this algorithm lies in its bee transformation capabilities. For instance, a scout bee could transform to an employed bee once it (the same scout bee) is involved in harvesting the food source and vice versa.

Generally, the bees can change their statuses depending on the needs of the algorithm at a particular point in time. In this algorithm, the food source represents a solution to the optimization problem. The volume of nectar in a food source represents the quality (fitness) of the solution. Moreover, each employed bee is supposed to exploit only one food source, meaning that the number of employed bees is the same as the number of food sources. The scout bees are always exploring for new food sources ${\vec{v}}_{m}$ with higher nectar quantity and/or quality (fitness) ${\vec{x}}_{m}$ within the neighbourhood. The bees evaluate the nectar fitness using

(7) $$\begin{array}{c} v_{mi}=x_{mi}+\emptyset_{mi}\left(\;x_{mi}-x_{ki}\;\right), \end{array}$$

where *i* is a randomly chosen parameter index; *∅*_*mi*_ is a random number within a given range; *x*_*ki*_ is a food source.

The quality (fitness) of a solution ${fit}_{m}(\vec{x_{m}})$ is calculated using the following equation:

(8) $$\begin{array}{c} {fit}_{m}\left(\;\vec{x_{m}}\;\right)=\left\{\begin{array}{cc} \frac{1}{1+f_{m}\left(\vec{x_{m}}\right)}x, & \text{if }f_{m}\left(\vec{x_{m}}\right)>0\\ 1+f_{m}\left(\vec{x_{m}}\right), & \text{if }f_{m}\left(\vec{x_{m}}\right)<0. \end{array}\right. \end{array}$$

From the foregoing discussion, it is clear that there is slight similarity between (5) in PSO and (7) in ABO since each algorithm subtracts a variable from the personal and individual bests of the particles/buffalos. For PSO, the subtracted variable is the present position and for ABO, it is the immediate-past explored location (the* waaa* values, *w*.*k*). However, the two equations are different in several respects: while the PSO uses *x* (being the constriction factor) or *ω* (as inertia factor, in some versions of the PSO), there are no such equivalents in ABO. Moreover, while the PSO employs random numbers (*U*_1_ and *U*_2_), ABO does not use random numbers, only learning parameters. Also, PSO uses acceleration coefficients (*∅*_1_ and *∅*_2_); ABO does not. In the case of the ABC, even though it employs the same search technique in arriving at solutions, the algorithm procedures are quite different.

### 2.4. Information Propagation

**Figure 1:**
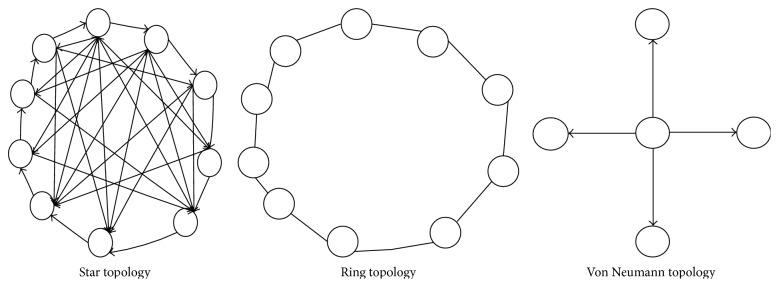
Information propagation topologies.

In searching for solutions to an optimization problem, the ACO employs the path construction technique while the PSO, ABC, and the ABO use the path improvement technique. However, while the PSO uses the Von Neumann (see Figure 1) as its best technique for information propagation [21], the ACO obtains good results using the ring topology [22] and the ABO uses the star topology which connects all the buffalos together. The Von Neumann topology enables the particles to connect to neighboring particles on the east, west, north, and south. Effectively, a particular particle relates with the other four particles surrounding it. The ABO employs the star topology such that a particular buffalo is connected to every other buffalo in the herd. This enhances ABO's information dissemination at any particular point in time.

## 3. African Buffalo Optimization Algorithm

In using the ABO to proffer solutions in the search space, the buffalos are first initialized within the herd population and are made to search for the global optima by updating their locations as they follow the current best buffalo *bg*max in the herd. Each buffalo keeps track of its coordinates in the problem space which are associated with the best solution (fitness) it has achieved so far. This value is called *bp*max.*k* representing the best location of the particular buffalo in relation to the optimal solution. The ABO algorithm follows this pattern: at each step, it tracks the dynamic location of each buffalo towards the *bp*max.*k* and *bg*max depending on where the emphasis is placed at a particular iteration. The speed of each animal is influenced by the learning parameters.

### 3.1. ABO Algorithm

The ABO algorithm is presented below:

(1) Initialization: randomly place buffalos to nodes at the solution space.
(2) Update the buffalos fitness values using
  (9) m.k+1=m.k+lp1bgmax−w.k+lp2bpmax.k−w.k,
 where *w*.*k* and *m*.*k* represent the exploration and exploitation moves, respectively, of the *k*th buffalo (*k* = 1,2,…, *N*); *lp*1 and *lp*2 are learning factors; *bg*max is the herd's best fitness and *bp*max.*k* the individual buffalo *k*'s best found location.
(3) Update the location of buffalo *k* (*bp*max.*k* and *bg*max) using
  (10) $$\begin{array}{c} w.k+1=\frac{\left(w.k+m.k\right)}{\pm 0.5}. \end{array}$$
(4) Is *bg*max updating? Yes, go to (5). No, go to (2).
(5) If the stopping criteria are not met, go back to algorithm step (3); else go to (6).
(6) Output best solution.

A closer look at the algorithm (the ABO algorithm) reveals that (9) which shows the democratic attitude of the buffalos has three parts: the first *m*.*k* represents the memory of the buffalos past location. The buffalo has innate memory ability that enables it to tell where it has been before. This is crucial in its search for solutions as it helps it to avoid areas that produced bad results. The memory of each buffalo is a list of solutions that can be used as an alternative for the current local maximum location. The second *lp*1(*bg*max − *w*.*k*) is concerned with the caring or cooperative part of the buffalos and is a pointer to the buffalo's social and information-sharing experience and then the third part *lp*2(*bp*max.*k* − *w*.*k*) indicates the intelligence part of the buffalos. So the ABO exploits the memory, caring intelligent capabilities of the buffalos in the democratic equation (9). Similarly, (10) is the waaa vocalization equation that propels the animals to move on to explore other environments as the present area has been fully exploited or is unfavourable for further exploration and exploitation.

### 3.2. Highlights of the ABO Algorithm

They are as follows:

(1) Stagnation handling through regular update of the best buffalo *bg*max in each iteration.
(2) Use of relatively few parameters to ensure speed fast convergence.
(3) A very simple algorithm that require less than 100 lines of coding effort in any programming language.
(4) Ensuring adequate exploration by tracking the location of best buffalo (*bg*max) and each buffalo's personal best location (*bp*max.*k*).
(5) Ensuring adequate exploitation through tapping into the exploits of other buffalos *lp*1(*bg*max − *w*.*k*).

### 3.3. Initialization

The initialization phase is done by randomly placing the *k*th buffalo in the solution space. For initialization, some known previous knowledge of the problem can help the algorithm to converge in less iterations.

### 3.4. Update Speed and Location

In each iteration, each buffalo updates its location according to its former maximum location (*bp*max) and some information gathered from the exploits of the neighboring buffalos. This is done using (9) and (10) (refer to the ABO algorithm steps (2) and (3) above). This enables the algorithm to track the movement of the buffalos in relation to the optimal solution.

## 4. Using ABO to Solve the Travelling Salesman's Problem

The ABO has the advantage of using very simple steps to solve complex optimization problems such as the TSP. The basic solution steps are as follows:

(a) Choose, according to some criterion, a start city for each of the buffalos and randomly locate them in those cities. Consider
  (11) Pab=wlp1abmlp2ab∑i=1nwlp1abmlp2abab=±0.5.
(b) Update buffalo fitness using (9) and (10), respectively.
(c) Determine the *bp*max.*k* and max.
(d) Using (11) and heuristic values, probabilistically construct a buffalo tour by adding cities that the buffalos have not visited.
(e) Is the *bg*max updating? Go to (f). No, go to (a).
(f) Is the exit criteria reached? Yes, go to (g). No, return to (b).
(g) Output the best result.

Here, *lp*1 and *lp*2 are learning parameters and are 0.6 and 0.4, respectively, *ab* takes the values of 0.5 and −0.5 in alternate iterations, *m* is the positive reinforcement alert invitation which tells the animals to stay to exploit the environment since there are enough pastures, and *w* is the negative reinforcement alarm which tells the animals to keep on exploring the environment since the present location is not productive. For buffalo *k*, the probability *p* ^*k*^ of moving from city *j* to city *k* depends on the combination of two values, namely, the desirability of the move, as computed by some heuristic indicating the previous attractiveness of that move and the summative benefit of the move to the herd, indicating how productive it has been in the past to make that particular move. The denominator values represent an indication of the desirability of that move.

### 4.1. ABO Solution Mechanism for the TSP

The ABO applies the Modified Karp Steele algorithm in its solution of the Travelling Salesman's Problem [23]. It follows a simple solution step of first constructing a cycle factor *F* of the cheapest weight in the *K* graph. Next, it selects a pair of edges taken from different cycles of the *K* graph and patch in a way that will result in a minimum weight. Patching is simply removing the selected edges in the two cycle factors and then replacing them with cheaper edges and in this way forming a larger cycle factor, thus, reducing the number of cycle factors in graph *K* by one. Thirdly, the second step is repeated until we arrive at a single cycle factor in the entire graph *K*. This technique fits into the path improvement technique description [24]. ABO overcomes the problem of delay in this process through the use of two primary parameters to ensure speed, namely, *lp*1 and *lp*2, coupled with the algorithm keeping a track of the route of the *bg*max as well as *bp*max.*k* in each construction step.

## 5. Experiments and Discussion of Results

In this study, the ABO was implemented on three sets of symmetric TSP datasets and a set of asymmetric TSP (ATSP) ranging from 48 to 14461 cities from TSPLIB95 [25]. The first experiment was concerned with the comparison of the performance of ABO in TSP instances with the results obtained from a recent study [26] involving Berlin52, St70, Eil76, Pr76, KroA100, Eil101, Ch150, and Tsp225. The second set of experiments was concerned with testing ABO's performance with another recently published study [27] on Att48, St70, Eil76, Pr152, Gil262, Rd400, Pr1002, D1291, Fn14461, and Brd14051. The third experiment examined ABO's performance in asymmetric TSP instances. The fourth set of experiments involved comparing ABO results with those obtained using some popular Artificial Neural Networks methods [28]. The TSP benchmark datasets are presented in Table 1.

### 5.1. Experimental Parameters Setting

For the sets of experiments involving PSO, the parameters are as follows: population size: 200; iteration (*T*_max_): 1000; inertia weight: 0.85; *C*_1_: 2; *C*_2_: 2 rand1(0,1)  rand2(0,1). For the HPSACO, the experimental parameters are as follows: population: 200; iteration (*T*_max_): 1000; inertia weight: 0.85; *C*_1_: 2; *C*_2_: 2; ants (*N*): 100; pheromone factor (*α*): 1.0; heuristic factor (*β*): 2.0; evaporation coefficient (*ρ*): 0.05; pheromone amount: 100. For the experiments involving other algorithms, in this study, Table 2 contains the details of the parameters used.

### 5.2. Experiment Analysis and Discussion of Results

The experiments were performed using MATLAB on Intel Duo Core i7-3770 CPU, 3.40 ghz with 4 GB RAM. The experiments on the asymmetric Travelling Salesman's Problems were executed on a Pentium, Duo Core, 1.80 Ghz processor and 2 GB RAM desktop computer. Similarly, the experiments on the Artificial Neural Networks were executed using Microsoft Virtual C++, 2008, on an Intel Duo Core, i7, CPU. To authenticate the veracity of the ABO algorithm in solving the TSP, we initially carried out experiments on eight TSP cases. The city-coordinates data are available in [29]. The results are presented in Table 3.

In Table 3, the “Average Value” refers to the average fitness of each algorithm, and the “relative error” values are obtained by calculating

(12) $$\begin{array}{c} \left(\;\frac{\text{Average value}-\text{Best value}}{\text{Best value}}\;\right)\times 100. \end{array}$$

As can be seen in Table 3, the ABO outperformed the other algorithms in all test instances under investigation. The ABO, for instance, obtained the optimal solution to Berlin52 and Eil76. No other algorithm did. Besides this, the ABO obtained the nearest-optimal solution to the remaining TSP instances compared to any other algorithm. In terms of the average results obtained by each algorithm, the ABO still has the best performance. It is rather surprising that the Hybrid Algorithm (HA) which uses a similar memory matrix like the ABO could not pose a better result. This is traceable to the use of several parameters since the HA is a combination of the ACO and the ABC: the two algorithms that have some of the largest numbers of parameters to tune in order to obtain good solutions.

The dominance of the ABO can also be seen in the use of computer resources (CPU time) where it is clearly seen that the ABO is the fastest of all four algorithms. In Berlin52, for instance, the ABO is 58,335 times faster than the ACO; 1,085 times faster than the ABC; 30,320 times faster than the Hybrid Algorithm (HA). This trend continues in all the test cases under investigation. To solve all the TSP problems here, it took ABO a cumulative time of 0.279 seconds to ACO's 7456 seconds; ABC's 43.11 seconds; and HA's 3362.27 seconds. The speed of the ABO is traceable to effective memory management technique since the ABO uses the path improvement technique as against the ACO that uses the slower path construction method. The difference in speed with the ABC that uses similar technique with ABO is due to the ABC's use of several parameters. The speed of the HA could have been affected by the combination of the path construction and path improvement techniques coupled with the use of several parameters.

### 5.3. ABO and Other Algorithms

Encouraged by the extremely good performance of the ABO in the first experiments, more experiments were performed and the results obtained compared with those from PSO, ACO, and HPSACO from another recently published study [27]. The HPSACO is a combination of three techniques, namely, the Collaborative Strategy, Particle Swarm Optimization, and the Ant Colony Optimization algorithms. The datasets used in this experiment are from the TSPLIB95 and they are Att48, St70, Eil76, Pr152, Gil262, Rd400, Pr1002, D1291, Fn14461, and Brd14051. The results are presented in Table 4.

Table 4 further underscores the excellent performance of ABO. The ABO obtained the optimal solution to Eil76 and Gil262 and very near-optimal solution in the rest of test cases. It is obvious from the relative error calculations that the ABO obtained over 99% accuracy in all the TSP instances here except the difficult rd100 where it obtained 95%. It is worthy of note that the 95% accuracy of the ABO is still superior to the performance of the other comparative algorithms in this TSP instance. The cumulative relative error of the ABO is 5.68% to the PSO's 61.83%, the ACO's 39.81%, and the HPSACO's 28.28%. Clearly, from this analysis, the ABO has a superior search capability. This is traceable to its use of relatively fewer parameters than most other Swarm Intelligence techniques. The controlling parameters of the ABO are just the learning parameters (*lp*1 and *lp*2).

In designing the ABO, the authors deliberately tried to avoid the “Frankenstein phenomena” [30], that is, a case of developing intricate techniques with several parameters/operators that, most times, the individual contributions of some of such parameters/operators to the workings of the algorithm are difficult to pinpoint. The ability to achieve this “lean metaheuristic” design (which is what we tried to do in designing the ABO) is a mark of good algorithm design [30].

## 6. ABO on Asymmetric TSP Test Cases

Moreover, we had to experiment on some ATSP test cases to verify the performance of the novel algorithm on such cases. The results obtained from this experiment using ABO are compared with the results obtained from solving some benchmark asymmetric TSP instances available in TSPLIB95 using the Randomized Arbitrary Insertion algorithm (RAI) [31], Ant Colony System 3-opt (ACS), Min-Max Ant System (MMAS), and Iterated Local Search (ILS) which is reported to be one of the best methods for solving TSP problems. These ATSP instances are ry48p, ft70, Kro124p, ftv70, p43, and ftv170 [32].

Table 5 shows the comparative results obtained by applying five different algorithms to solving a number of benchmark asymmetric TSP instances with the aim of evaluating the performance of the African Buffalo Optimization (ABO) algorithm. The first column lists the ATSP instances as recorded in the TSPLIB; the second column indicates the number of cities/locations/nodes involved in each instance; the third indicates the optimal solutions, then followed by the performances of the different algorithms in at most 50 test runs.

A quick look at the table shows the excellent performance of the ILS and the MMAS with both obtaining 100% results in the only four cases available in literature that they solved. The ABO performed very well achieving over 99% in all test cases but one. The ABO has approximately the same performance with RAI and the ACS obtaining about 99% optimal results in virtually all cases. It is significant to note, however, that the ABO performed better than ACS and RAI in ft70 which was said to be a difficult ATSP instance to solve in spite of its relatively small size [33].

Next, the authors examined the speed of each algorithm to achieve result since one of the aims of the ABO is to solve the problem of delay in achieving optimal solutions since speed is one of the hallmarks of a good algorithm [34]. In Table 6, we compare the speed of the ABO with those of the recently published Model Induced Max-Min Ant Colony Optimization (MIMM-ACO), Min-Max Ant System (MMAS) Cooperative Genetic Ant Systems (CGAS) [35], and RAI.

Table 6 displays the exceptional capacity of the novel African Buffalo Optimization (ABO) to obtain optimal or near-optimal solutions at record times. It took the ABO less than a second (precisely 0.95 seconds) to solve all the benchmark cases under investigation to MIMM-ACO's 232.09 seconds; MMAS's 208.88 seconds; CGAS' 452.927 seconds; and RAI's 323.4 seconds. Undoubtedly, the ABO outperformed the other algorithms in its quest to obtain results using very limited CPU time and resources. This brought about ABO's use of very few parameters coupled with straightforward fitness function. The exceptional speed of the ABO compared with its competitive ability to obtain very competitive results recommends the ABO as an algorithm of choice when speed is a factor.

## 7. Comparing ABO to Neural Network Solutions to TSP

Moreover, following the popularity and efficiency of Neural Networks in obtaining good solutions to the Travelling Salesman's Problem [36], the authors compared the performance of African Buffalo Optimization to the known solutions of some popular Neural Network algorithms. These are Angeniol's method, Somhom et al.'s method, Pasti and Castro's method, and Masutti and Castro's method. Comparative experimental figures are obtained from [28].

From the experimental results in Table 7, it can be seen that only the ABO obtained the optimal solutions in Eil51 and Eil76, in addition to jointly obtaining the optimal result with Masutti and Castro's method in Berlin52. Aside from these, ABO outperformed the other methods in getting near-optimal solutions in Bier127, KroA100, KroB100, KroB100, KroC100, KroD100, KroE100, Ch130, Ch150, KroA150, KroB150, KroA200, KroB150, KroB200 Rat575, rl1323, fl1400, fl1400, and Rat783. It was only in Eil101 that Masutti and Castro's method outperformed ABO. This is a further evidence that ABO is an excellent performer even when in competition with Neural Networks methods.

## 8. Conclusion

In general, this paper introduces the novel algorithm, the African Buffalo Optimization, and shows its capacity to solve the Traveling Salesman's Problem. The performance obtained from using the ABO is weighed against the performance of some popular algorithms such as the Ant Colony Optimization (ACO), Particle Swarm Optimization (PSO), Artificial Bee Colony Optimization (ABO), Min-Max Ant System (MMAS), and Randomized Insertion Algorithm (RAI); some hybrid methods such as Model Induced Max-Min Ant Colony Optimization (MIMM-ACO), Hybrid Algorithm (HA), and Cooperative Genetic Ant Systems (CGAS); and some popular Neural Networks-based optimization methods. In total, 33 TSP datasets cases ranging from 48 to 14461 cities were investigated and the ABO results obtained were compared with results obtained from 11 other optimization algorithms and methods. The results show the amazing performance of the novel algorithm's capacity to obtain optimal or near-optimal solutions at an incredibly fast rate. The ABO's outstanding performance is a testimony to the fact that ABO has immense potentials in solving optimization problems using relatively fewer parameters than most other optimization algorithms in literature.

Having observed the robustness of the ABO in solving the Travelling Salesman's Problems with encouraging outcome, it is recommended that more investigations be carried out to ascertain the veracity or otherwise of this new algorithm in solving other problems such as PID tuning, knapsack problem, vehicle routing, job scheduling, and urban transportation problems.

## Figures and Tables

**Table 1:** TSPLIB datasets.

1st experimental datasets	2nd experimental datasets	ATSP dataset	NN comparative datasets		
Berlin52	Att48	R48p	Eil51	KroA100 B	FL1400
St70	St70	Ft70	Eil76	KroA200	D1655
Eil76	Eil76	Kro124p	Eil101	KroB100	
Pr76	Pr152	Ftv70	Berlin52	KroB150	
Kroa100	Gil262	P43	Bier127	KroB200	
Eil101	Rd400	Ftv170	Ch130	KroC100	
Ch150	Pr1002		Ch150	KroD100	
Tsp225	D1291		Rd100	KroE100	
	Fnl4461		Lin105	Rat575	
	Brd14051		Lin318	RL1323	

**Table 2:** Experimental parameters.

ABO		ACO		ABC		HA		CGAS	
Parameter	Values	Parameters	Values	Parameters	Values	Parameters	Values	Parameter	Values
Population	40	Ants	*D* ^*∗*^	Population	*D* ^*∗*^	Population	*D* ^*∗*^	Generation	100
*m*.*k*	1.0	*β*	5.0	*ϕij*	rand(−1, 1)	*β*	5.0	*β*	2.0
*bg*max/*bp*max	0.6	*ρ*	0.65	*ωij*	rand(0, 1.5)	*ρ*	0.65	*ρ*	0.1
*lp*1/*lp*2	0.5	*α*	1.0	SN	NP/2	*α*	1.0	*Ro*	0.33
*w*.*k*	1.0	*Ǫ*	200	Limit	*D* ^*∗*^SN	*ϕij*	rand(−1, 1)	Crossover rate	1.0
N/A	—	*qo*	0.9	Max cycle number	500	*ωij*	rand(0, 1.5)	*qo*	0.9
N/A	—	N/A	—	Colony	50	SN	NP/2	*ϕr*	0.3
N/A	—	N/A	—	N/A	—	Limit	*D* ^*∗*^SN	*ϕρ*	0.2
N/A	—	N/A	—	N/A	—	Max cycle number	500	*τ*_min⁡_	*τ*_max⁡_/20
N/A	—	N/A	—	N/A	—	Colony	50	*τ*_max⁡_	1 − (1 − *ρ*)
N/A	—	N/A	—	N/A	—	*Ǫ*	200	N/A	—
N/A	—	N/A	—	N/A	—	*qo*	0.9	N/A	—
Total number of runs	50		50		50		50		50

**Table 3:** Comparative experimental result.

Problem	Number of cities	Optima	Method	Best	Mean	Rel. err. (%)	Time (s)
Berlin52			ABO	7542	7616	0%	0.002
			ACO	7548.99	7659.31	1.52	116.67
	52	7542	ABC	9479.11	10,390.26	37.72	2.17
			HA	7544.37	7544.37	0.03	60.64

St70			ABO	676	678.33	0.15	0.08
			ACO	696.05	709.16	4.73	226.06
	70	675	ABC	1162.12	1230.49	81.73	3.15
			HA	687.24	700.58	3.47	115.65

Eil76			ABO	538	563.04	0%	0.03
	76	538	ACO	554.46	561.98	3.04	271.98
			ABC	877.28	931.44	70.78	3.49
			HA	551.07	557.98	2.31	138.82

Pr76		108159	ABO	108167	108,396	0.007%	0.08
	76		ACO	115,166.66	116,321.22	7.55	272.41
			ABC	195,198.9	205,119.61	89.65	3.50
			HA	113,798.56	115,072.29	6.39	138.92

Kroa100		21282	ABO	21311	22163.8	0.4%	0.00
	100		ACO	22,455.89	22,880.12	7.49	615.06
			ABC	49,519.51	53,840.03	152.94	5.17
			HA	22,122.75	22,435.31	5.40	311.12

Eil101		629	ABO	640	640	1.7%	0.027
	101		ACO	678.04	693.42	7.96	527.42
			ABC	1237.31	1315.95	104.88	5.17
			HA	672.71	683.39	6.39	267.08

Ch150		6528	ABO	6532	6601	0.06%	0.032
	150		ACO	6648.51	6702.87	2.61	1387.65
			ABC	20,908.89	21,617.48	230.93	8.95
			HA	6641.69	6677.12	2.21	698.61

Tsp225		3916	ABO	3917	3982	0.03	0.09
	225		ACO	4112.35	4176.08	8.22	4038.75
			ABC	16,998.41	17,955.12	365.2792	16.68
			HA	4090.54	4157.85	7.74	2037.33

**Table 4:** Comparative optimal results.

TSP instance	Optima	ABO			PSO			ACO			HPSACO		
		Best	Avg	Err. %	Best	Avg	Err. %	Best	Avg	Err. %	Best	Avg	Err. %
att48	33522	33524	33579	0.16	33734	33982	0.63	33649	33731	0.62	33524	33667	0.16
st70	675	676	678.33	0.15	691.2	702.6	2.40	685.7	694.7	1.59	680.3	698.6	0.79
eil76	538	538	563.04	0.00	572.3	589.1	6.38	550.7	560.4	2.36	546.2	558.1	1.52
pr152	73682	73730	73990	0.07	75361	75405	2.28	74689	74936	1.37	74165	74654	0.66
gil262	2378	2378	2386	0.00	2513	2486	5.68	2463	2495	3.57	2413	2468	1.47
rd400	15281	15301	15304	5.00	16964	17024	11.01	16581	16834	8.51	16067	16513	5.14
pr1002	259045	259132	261608	0.03	278923	279755	7.67	269758	271043	4.14	267998	269789	3.46
d1291	50801	50839	50839	0.07	53912	54104	6.12	52942	53249	4.21	52868	52951	4.07
fnl4461	182566	182745	183174	0.10	199314	199492	9.17	192964	194015	5.70	191352	192585	4.81
brd14051	469385	469835	479085	0.10	518631	519305	10.49	505734	511638	7.74	498471	503594	6.20

**Table 5:** Comparative results.

TSP cases	Number of cities	Optimal values	ABO			RAI			MMAS			ACS			ILS		
			Best	Avg	Rel. error	Best	Avg	Rel. error	Best	Avg	Rel. error	Best	Avg	Rel. error	Best	Avg	Rel. error
Ry48p	48	14422	14440	14455	0.12%	14422	14543.20	0%	14422	14422	0%	14422	14565.45	0%	14422	14422	0%
Ft70	70	38673	38753	38870.5	0.21%	38855	39187.75	0.47%	38673	38687	0%	38781	39099.05	0.28%	38673	38687	0%
Kro124p	100	36230	36275	36713	0.12%	36241	36594.23	0.04%	36230	36542	0%	36241	36857	0.04%	36230	36542	0%
Ftv70	71	1950	1955	1958.5	0.26%	1950	1968.44	0%	—	—	—	—	—	—	—	—	—
P43	43	5620	5645	5698	0.44%	5620	5620.65	0%	—	—	—	—	—	—	—	—	—
Ftv170	171	2755	2795	2840.5	1.45%	2764	2832.74	0.33%	2755	2755	0%	2774	2826	0.69%	2755	2756	0%

**Table 6:** Comparative speed of algorithms.

TSP cases	Number of cities	ABO	MIMM-ACO	MMAS	CGAS	RAI
		Avg time (secs)	Avg time (secs)	Avg time (secs)	Avg time (secs)	Avg time (secs)
Ry48p	48	0.07	7.83	7.97	12.35	1.598
Ft70	70	0.05	9.85	10.15	15.32	7.068
Kro124p	100	0.08	33.25	23.4	78.52	30.34
Ftv70	71	0.09	64.53	61.25	69.64	7.376
P43	43	0.1	8.35	9.38	0.997	0.997
Ftv170	171	0.65	108.28	96.73	276.1	276.1

**Table 7:** ABO versus NN results.

TSP instance	Optima	ABO		Angeniol's method		Somhom et al.'s method		Pasti and Castro's method		Masutti and Castro's method	
		Best	Mean	Best	Mean	Best	Mean	Best	Mean	Best	Mean
eil51	426	426	427	432	442.90	433	440.57	429	438.70	427	437.47
eil76	538	538	563.04	554	563.20	552	562.27	542	556.10	541	556.33
eil101	629	640	640	655	665.93	640	655.57	641	654.83	638	648.63
berlin52	7542	7542	7659.31	7778	8363.70	7715	8025.07	7716	8073.97	7542	7932.50
bier127	118282	118297	118863	120110	128920.33	119840	121733.33	118760	121780.33	118970	120886.33
ch130	6110	6111	6307.14	6265	6416.80	6203	6307.23	6142	6291.77	6145	6282.40
ch150	6528	6532	6601	6634	6842.80	6631	6751	6629	6753.20	6602	6738.37
rd100	7910	7935	7956	8088	8444.50	8028	8239.40	7947	8253.93	7982	8199.77
lin105	14379	14419	14452.7	14999	16111.37	14379	14475.60	14379	14702.23	14379	14400.17
lin318	42029	42101	42336	44869	45832.83	43154	43922.90	42975	43704.97	42834	43696.87
kroA100	21282	21311	22163.8	23009	24678.80	21410	21616.77	21369	21868.47	21333	21522.73
kroA150	26524	26526	27205	28948	29960.90	26930	27401.33	26932	27346.43	26678	27355.97
kroA200	29368	29370	30152	31669	33228.33	30144	30415.67	29594	30257.53	29600	30190.27
kroB100	22141	22160	22509	24026	25966.40	22548	22622.50	22596	22853.60	22343	22661.47
kroB150	26130	26169	26431	27886	29404.53	26342	26806.33	26395	26752.13	26264	26631.87
kroB200	29437	29487	29534	32351	33838.13	29703	30286.47	29831	30415.60	29637	30135.00
kroC100	20749	20755	20881.7	22344	23496.13	20921	21149.87	20915	21231.60	20915	20971.23
kroD100	21294	21347	21462	23076	23909.03	21500	21845.73	21457	22027.87	21374	21697.37
kroE100	22068	22088	22702	23642	24828.03	22379	22682.47	22427	22815.50	22395	22715.63
rat575	6773	6774	6810	8107	8301.83	7090	7173.63	7039	7125.07	7047	7115.67
rat783	8806	8811	8881.75	10532	10721.60	9316	9387.57	9185	9326.30	9246	9343.77
rl1323	270199	270480	278977	293350	301424.33	295780	300899.00	295060	300286.00	300770	305314.33
fl1400	20127	20134	20167	20649	21174.67	20558	20742.60	20745	21070.57	20851	21110.00
d1655	62128	62346	62599.5	68875	71168.07	67459	68046.37	70323	71431.70	70918	72113.17
